# Unilateral Blinking: Insights from Stereo-EEG and Tractography

**DOI:** 10.1007/s10548-021-00865-x

**Published:** 2021-08-16

**Authors:** Elisabeth Kaufmann, Joanna Bartkiewicz, Nicholas Fearns, Katharina Ernst, Christian Vollmar, Soheyl Noachtar

**Affiliations:** Epilepsy Center, Department of Neurology, University of Munich, Marchioninistr. 15, 81377 Munich, Germany

**Keywords:** Eye closure, Epilepsy, DTI, Intracranial EEG

## Abstract

To study the neuroanatomical correlate of involuntary unilateral blinking in humans, using the example of patients with focal epilepsy. Patients with drug resistant focal epilepsy undergoing presurgical evaluation with stereotactically implanted EEG-electrodes (sEEG) were recruited from the local epilepsy monitoring unit. Only patients showing ictal unilateral blinking or unilateral blinking elicited by direct electrical stimulation were included (n = 16). MRI and CT data were used for visualization of the electrode positions. In two patients, probabilistic tractography with seeding from the respective electrodes was additionally performed. Three main findings were made: (1) involuntary unilateral blinking was associated with activation of the anterior temporal region, (2) tractography showed widespread projections to the ipsilateral frontal, pericentral, occipital, limbic and cerebellar regions and (3) blinking was observed predominantly in female patients with temporal lobe epilepsies. Unilateral blinking was found to be associated with an ipsilateral activation of the anterior temporal region. We suggest that the identified network is not part of the primary blinking control but might have modulating influence on ipsilateral blinking by integrating contextual information.

## Introduction

Blinking is essential for facial expression (emotional blinking) and ocular protection (reflective blinking), but can also occur voluntarily, or as conditioned response. Its multifaceted importance demands a detailed understanding of its neuroanatomical control.

Blinking arises mainly by a paroxysmal contraction of the orbicularis oculi muscle, is typically bilateral and of a wink-wise character (Ponder and Kennedy 1927). The contraction of the orbicularis oculi muscle is mediated by repetitive electric discharges originating of the facial nerve or its motor nucleus (Holstege et al. 1986; Jacome 1999). Its supranuclear control, however, is still incompletely understood. *Indirect information* is obtained by lesional or neuroanatomical studies, neuronal tracing, functional neuroimaging studies, as well as trans-/extracranial stimulation experiments. In primates, facial motor function is represented by at least six cortical areas. These include the primary motor (M1), the supplementary motor (M2), the dorsal lateral (LPMCd) and ventral lateral premotor (LPMCv), as well as the rostral (M3) and caudal cingulate (M4) motor cortices (Morecraft et al. 2001; Gong et al. 2005a). Secondary cortical input is attributed to the frontal eye field, the somatosensory cortex, the visual cortex, the cerebellum, the superior temporal lobe, as well as limbic structures (Morecraft et al. 2001; Gong et al. 2005a). These wide-spread cortical projections and their cortico-cortical interconnections imply that a complex supranuclear network is involved in the context-related regulation of blinking (Muakkassa and Strick 1979; Morecraft et al. 2004). This hypothesis is further supported by phenomena like the volitional or emotional facial paralysis, i.e. an impaired voluntary activation of facial muscles but normal activation with emotion and vice versa. These phenomena are typically observed after localized brain damage in the anterior opercular region or the midline cortex, the insula, the thalamus, the striatocapsular region or the pons (Foix et al. 1926; Alajouanine and Thurel 1933; Borod et al. 1988; Mao et al. 1989; Hopf et al. 1992, 2000; Bakar et al. 1998; Urban et al. 1998; Holstege 2002).

The gold standard to obtain *direct information* about the neuroanatomical network of blinking contral in vivo would be electrical cortical stimulation and intracranial electrophysiological recording. Such data, though, is as yet limited for blinking in humans. Here we report on epilepsy patients in whom unilateral blinking was induced by ipsilateral cortical stimulation using stereotactically implanted electroencephalography (EEG) electrodes.

## Methods

This study complies with the ethical guidelines of the University of Munich and was approved by the institutional review board. All patients gave written informed consent to the scientific use of their clinically acquired and anonymized data.

### Participants

The recruitment was started after the first incidental finding of stimulation induced unilateral blinking in 01/2015. Patients with drug resistant focal epilepsy and stimulation induced unilateral blinking were then prospectively recruited from the epilepsy monitoring unit at the University of Munich until 12/2020. Only patients with stereo-EEG (sEEG) electrodes in the temporal and/or frontal lobe and available available documentation of the intracranial stimulation results were considered for the study (n = 60).

### Video-EEG-Monitoring

All patients have undergone invasive evaluation with up to twelve stereotactically implanted Spencer depth electrodes (Ad-Tech Medical Instrument Corporation, Racine, USA) with a diameter of 0.86 mm and 4–14 contacts, each 5–10 mm apart and of 5 mm length. Stereo-EEG was recorded using XLTEK Neuroworks software (Natus Medical Incorporated, San Carlos, USA) and an XLTEK EMU128FS amplifier with a sampling rate of 1000 Hz and 16 bit A-D conversion. In addition, surface electrodes were placed on the accessible skin surface using the international 10–20 system of electrode placement. Epilepsy syndromes were classified in an interdisciplinary patient management conference based on the available EEG data, seizure semiology, neuropsychological test results, as well as the functional and structural imaging data.

### Electrical Intracranial Stimulation

After successful recording of seizures, electrical stimulation was performed in a referential (monopolar mode) using the same stereotactically implanted depth electrodes. An electrode contact in a non-eloquent and non-epileptic brain area was thereby used as reference. Stimulation was performed on all electrode contacts within as well as in spatial proximity of the planned resection area. Therefore, not all stimulated contacts have been within cerebral gray matter, but also within white matter. Electrical stimulation within white matter might more likely lead to current conduction and thus remote clinical effects. Stimulation parameters were set to biphasic stimulation with a frequency of 50 Hz, a fixed pulse width of 300 µs, a maximum duration of five seconds, and a stimulation intensity of 1–15 mA. The latter was gradually increased by steps of 1–2 mA until the maximum stimulation intensity of 15 mA was reached or any clinical symptoms or side effects occurred. In principle, elicited symptoms have a high localizing value if they occur at low stimulation amplitudes (< 8 mA) and are more unspecific if they occur only at higher stimulation amplitudes. To minimize the risk of seizures, stimulation was only performed with antiseizure medication.

### Electrode Localization

The locations of the intracranial electrodes were precisely determined using co-registered preoperative magnetic resonance imaging (MRI) and postoperative computed tomography (CT) scans. For group visualization, electrode positions were additionally normalized to MNI space, (Montreal Neurological Institute), based on T1 MRI to template registration using SPM software (https://www.fil.ion.ucl.ac.uk/spm/) and mirroring right hemispheric contacts to the left hemisphere. Amira Software (Thermo Fisher Scientific, Waltham, MA, USA) was used for 3-dimensional visualization.

### Tractography

In order to visualize the fiber bundles originating from the stimulation site, probabilistic fiber tracking was performed whenever diffusion tensor imaging (DTI) data was available (acquired on a 3 T GE Signa HDx Scanner using 64 diffusion-weighted directions, b-value of 1000 s/mm^2^, 60 axial slices with 2.4 mm slice thickness, 96 × 96 in-plane matrix, 220 mm field of view, TR 16,000 ms, TE 90.2 ms, flip angle 90°, SENSE factor 2 for parallel imaging). The DTI data was resampled to isotropic 1 mm voxel size and then corrected for eddy current distortions and skull-stripped using the FMRIB Software Library Version 4.1.6 (FSL; http://fsl.fmrib.ox.ac.uk), FSL dtifit was used to create fractional anisotropy (FA) maps. Whole brain tractography was performed using a high-angular resolution diffusion imaging (HARDI) approach (http://trackvis.org/blog/tag/diffusion-toolkit/). Tracking used a 2nd-order Runge Kutta propagation algorithm with an angular threshold of < 35° between neighboring voxels. The termination criteria of the tracking were either fiber length of more than 200 mm or reaching a voxel with an FA value < 0.1. Seeding was implemented using a 5 mm big sphere around the electrode contact of interest. For visualization, only 10% of the reconstructed fiber tracks are shown.

### Statistical Analysis

Mean and standard deviation were calculated for quantitative parameters. Relative frequencies are presented as total numbers as well as percentages.

## Results

### Stimulation Induced Eyelid Contraction

Stimulation induced unilateral blinking or eyelid closure was observed in 16/60 (26.7%) patients (5 men, 11 females; 31.56 ± 11.04 years of age; age at disease onset: 18.50 ± 11.57 years) The clinical details of the patients are summarized in Table 1.

**Table 1:** Patient characteristics

ID	Sex	Age	Epilepsy type	MRI	Prior surgery
1	f	40	TLE right	Hippocampal asymmetry right > left	No
2	f	55	TLE right	Right hippocampal swelling	No
3	m	19	TLE left	Ganglioglioma left hippocampal	Microresection of ganglioglioma left hippocampal
4	f	52	TLE left	Ependymoma left fronto-temporal	No
5	m	41	TLE left	Reduced brain volume left temporal, temporal herniation of the frontal lobe, FCD IIIa	No
6	f	22	multifocal	Gliosis left parahippocampal	Resection medulloblastoma
7	f	37	TLE left	Schizencephaly	No
8	f	22	TLE right	Nonlesional	No
9	f	31	Insular left	Nonlesional	No
10	m	13	TLE left	Cystic glyotic lesion left temporopolar	Resection plexus carcinoma
11	m	29	FTLE right	Nonlesional	No
12	f	31	TLE left	HS, cavernoma left frontal	No
13	f	24	TLE right	FCD	Anterior temporal resection
14	f	32	TLE right	HS	No
15	m	25	FLE right	Postcontusional defect right frontal	No
16	f	32	FTLE right	HS, FCD right frontal	No

*f* female, *FCD* focal cortical dysplasia, *FLE* frontal lobe epilepsy, FTLE frontotemporal lobe epilepsy, *HS* hippocampal sclerosis, *m* male, *TLE* temporal lobe epilepsy

Ipsilateral eyelid contraction was provoked by stimulation in the anterior temporal lobe, including the temporal pole, uncus, amygdala, hippocampus, parahippocampal and fusiform gyrus (left: n = 6; right: n = 9) using an amplitude of 1 to 14 mA, as well as in one patient by stimulation in the fronto-orbital region (left: n = 1) using an amplitude of 4 mA. The latter patient showed a herniation of the fronto-orbital cortex into the middle cranial fossa most likely resulting in an anterior temporal stimulation effect. The electrical stimulation of the amygdala/uncus and the anterior part of the parahippocampal/fusiform gyrus were associated with the highest blinking rates, i.e. blinking was observed in 9 out of 47 (19.1%) or 9 out of 48 (18.8%) stimulations, respectively. Lower rates were observed for the anterior hippocampus (5/45; 11.1%) and the posterior fusiform gyrus (1/46; 2.2%). Ipsilateral blinking did not occur upon insular (n = 49), fronto-orbital (n = 47) and parietal stimulations (n = 15).

**Fig. 1:**
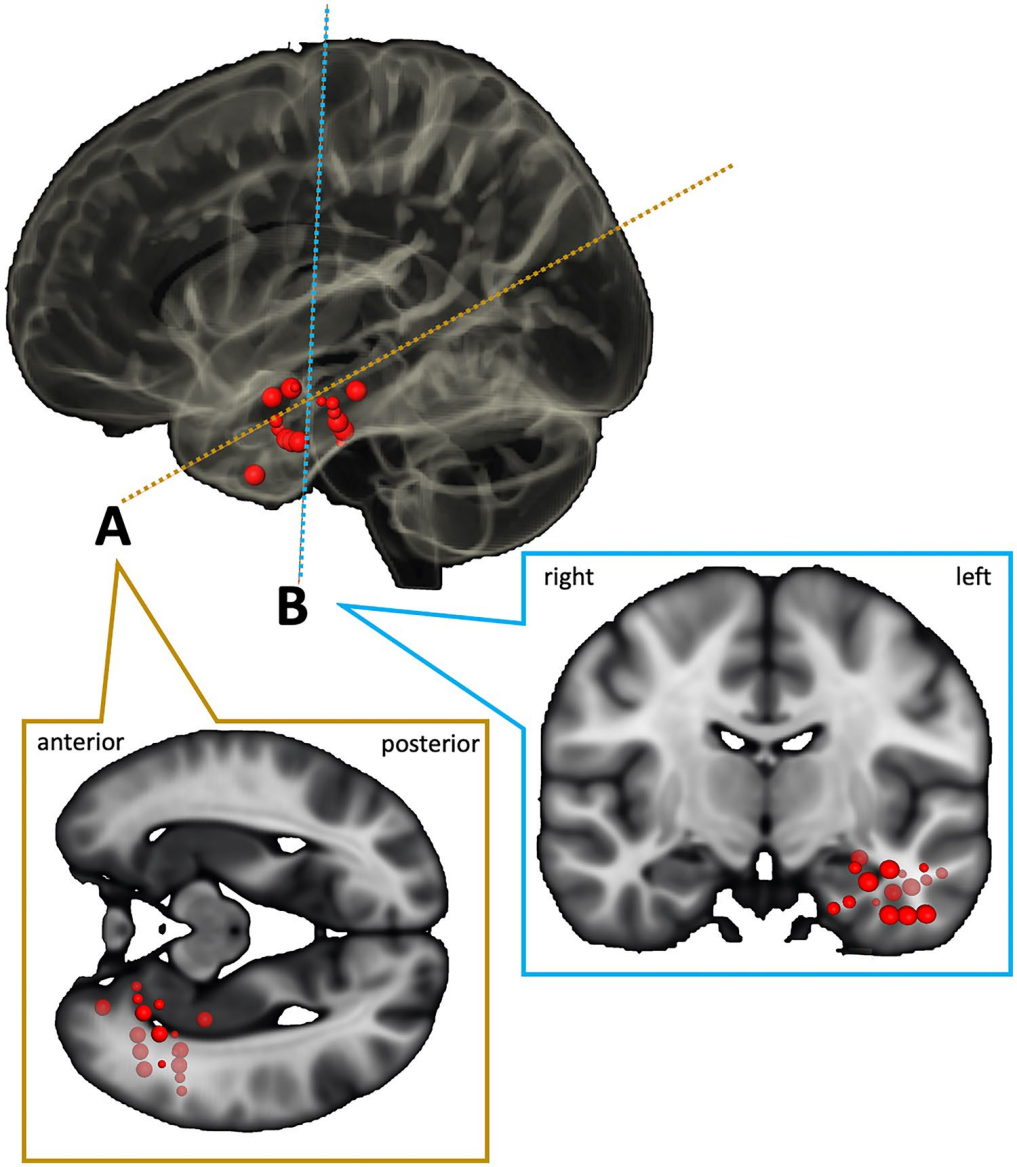
Grouped visualization of contacts associated with unilateral blinking. The figure summarizes the anatomical location of the SEEG electrode contacts (red dots) that were associated with unilateral blinking or tonic eye closure upon their electrical stimulation in 8 representative patients. The size of the red dots represents the specificity of the observed effect, i.e. small dots representing electrodes where blinking was elicited only at higher stimulation amplitudes > 12 mA and the biggest dots representing the most specific effects that occurred already at < 4 mA. The individual contact locations were determined via co-registration of the postop CT and preoperative MRI scans and transferred to a common space (MNI space). Contacts located within the right hemisphere were mirrored to the left hemisphere. **A** Sagittal cut with hippocampal angulation. **B** Coronal cut through the anterior temporal lobe. The dashed lines in the uppermost figure represent the respective section planes (Color figure online)

Low stimulation amplitudes were sufficient to elicit the eyelid contraction if the respective electrode contact was within the gray matter, whereas higher stimulation amplitudes were required when stimulating in white matter regions. The eyelid contraction always began simultaneously with the stimulation onset (n = 16) and stopped isochronal with stimulation cessation (n = 15). In one patient (ID1), electrical stimulation with 3 mA in the right parahippocampal gyrus elicited unilateral blinking, which evolved into an epileptic seizure three times. The respective electrode contacts were located in close vicinity to the seizure onset zone. In five patients, post-stimulation after-discharges were elicited at higher stimulation amplitudes (> 9 mA) but blinking always ceased at the end of the stimulation and did not continue during the post-stimulation discharges. A grouped visualization of the identified fronto-temporal SEEG contacts of 8 representative patients is shown in Fig. 1.

Seven patients had a predominant clonic eyelid contraction during temporal stimulation. The stimulation site was in close vicinity to their epileptic zone or even elicited a habitual seizure. The other nine patients, in contrast, showed a tonic eyelid contraction. In some of these patients we observed that lower stimulation amplitudes caused palpebral flutter of the eyelid before tonic contraction at higher stimulation amplitudes. The eyelid on the contralateral side, though, always remained unaffected.

In contrast, the other 44 patients with SEEG electrodes in the fronto-orbital and/or temporal region who did not reveal unilateral blinking upon stimulation did not significantly differ from the study cohort with regard to age, disease duration and epileptic hemispheres. However, they encompassed significantly less females (39% vs. 75%; p = 0.0190) and less patients with temporal lobe epilepsy (34% vs.68% p = 0.0212).

### Tractography

**Fig. 2:**
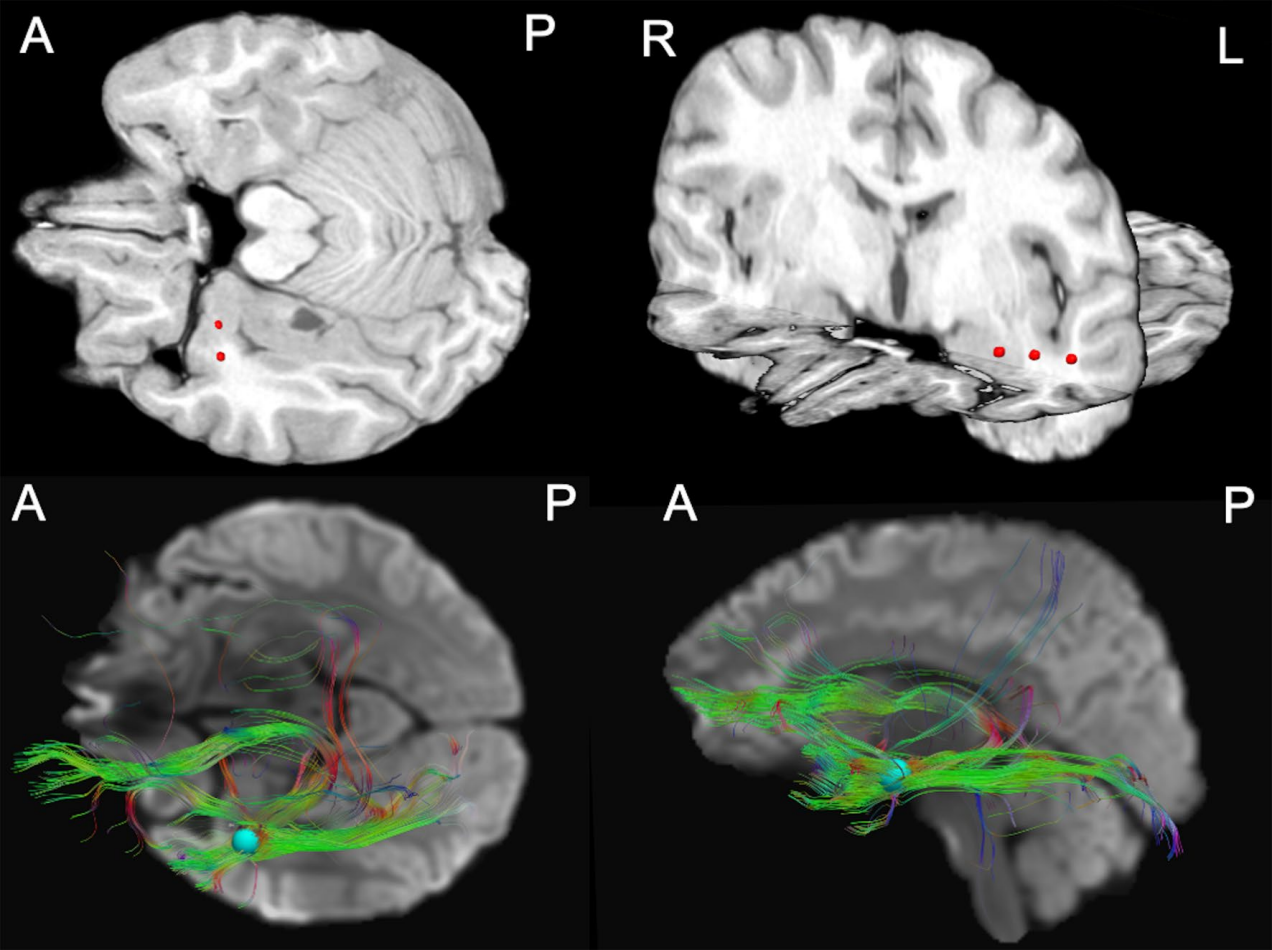
Tractography results. The upper row shows the location of the electrodes which were associated with unilateral blinking in one representative patient (ID3). The location of the stereo-EEG electrodes was determined by a co-registration of the preoperative MRI and postoperative CT scan.The lower row shows the HARDI fiber tracking results seeding from a 5 mm big sphere. The spheres were positioned around one of the electrodes shown in the 2D images above. For reasons of clearness, only 10% of the reconstructed fibers are shown. As background, average diffusion weighted imaging maps were used. *A* anterior, *L* left, *P* posterior, *R* right

Tractography was performed for two representative patients (ID 3; ID 5) with stimulation induced blinking. Seeding from the left anterior temporal region, where blinking was provoked by electrical stimulation in one patient (ID 3), revealed projections to the ipsilateral cerebellum, fornix and posterior temporal region besides the projections to the frontopolar and pericentral region. In the second patient (ID 5), blinking was induced by electrical stimulation in the herniated left basal fronto-orbital region, where tractography revealed connections not only to the ipsi- but also the contralateral mesial frontopolar region (Fig. 2).

## Discussion

### Cerebral Regions Associated with Unilateral Blinking

Our findings indicate an association of unilateral blinking/eyelid closure with activation of the ipsilateral anterior temporal region. Most frequently, unilateral blinking/eyelid closure was elicited upon electrical stimulation within the amygdala/uncus and fusiform/parahippocampal gyrus. This observation is in line with former reports on fronto-temporal seizure patterns in scalp-EEG recordings during ictal ipsilateral blinking (Benbadis et al. 1996; Henkel et al. 1999; Pestana and Gupta 2007; Kalss et al. 2013), as well as a parahippocampal activation during spontaneous blinking in functional MRTI (fMRI) investigations (Yoon et al. 2005). Of note, in monkeys and rats, retrograde neuronal tracing from the orbicularis oculi muscle revealed higher-order nerval connections spreading into the ipsilateral temporal lobe, i.e. the superior temporal sulcus or the temporal cortex (Morcuende et al. 2002; Gong et al. 2005a).

### Significance of Anterior Temporal Input to the Generation of Blinking

Presumably, the identified anterior temporal regions have no direct projections to the facial nucleus. It is more likely that they are part of a network of motor cortical projections that influences the primary motor pathway. Previous data suggests a context-dependent activation of this motor network, but the exact way of interaction of the different cortical components or its modulation are still not fully understood (Dimitrova et al. 2002; Yoon et al. 2005).

**Fig. 3:**
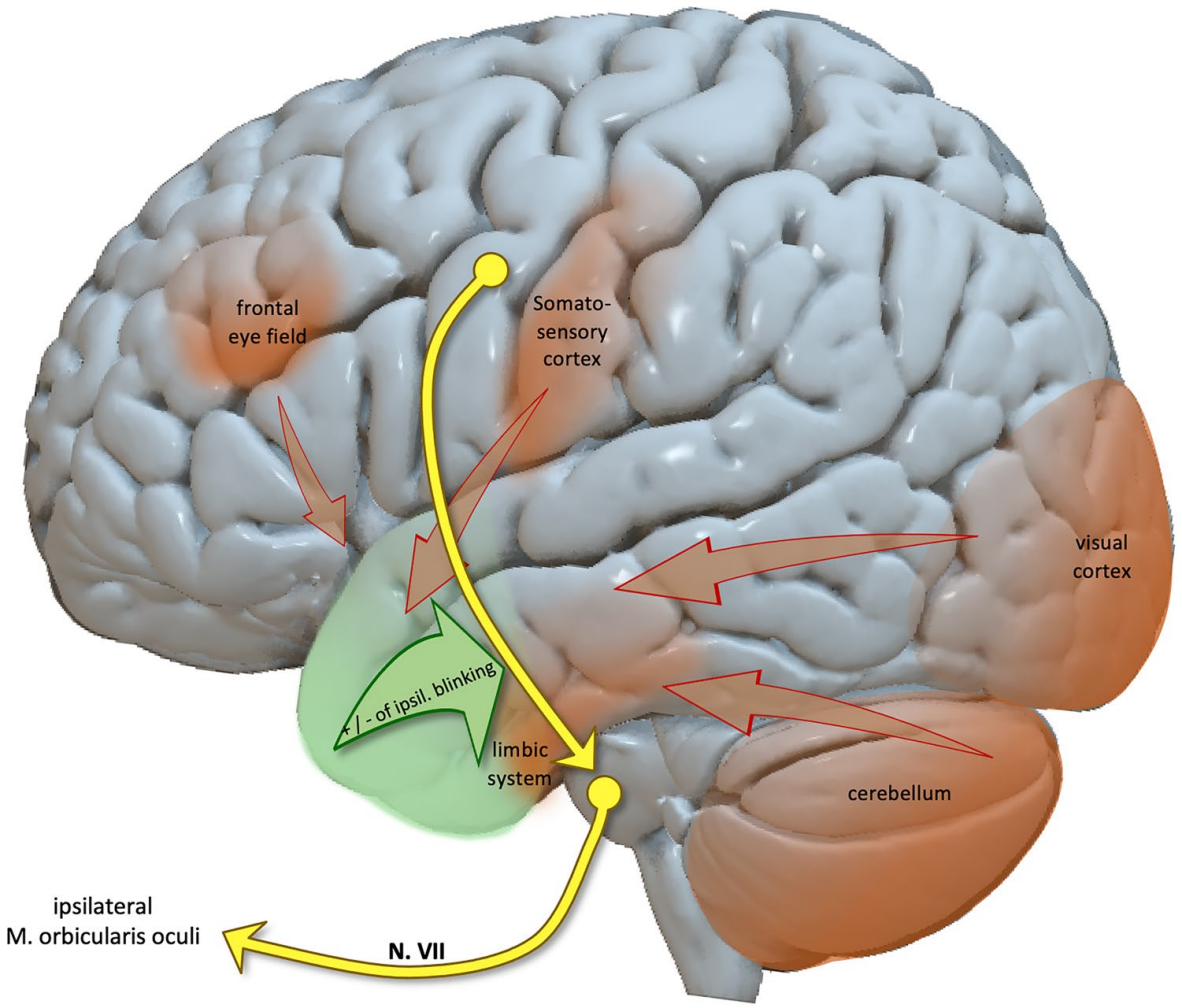
Hypothesized supranuclear facial motor network. The identified anterior temporal region (greenish) are supposed to integrate information from different secondary facial motor cortices (reddish). By integrating the emotional, visuospatial, and sensorial data, the anterior temporal structures might be able to modulate the primary facial motor projections in a context-dependent manner. In other words, the anterior temporal region might reinforce or inhibit the ipsilateral blinking response depending on e.g. the emotional state or facial sensations like trigeminal pain. An MNI surface template from the commercially available software package Lead DBS was used for the visualization (Horn et al. 2019) (Color figure online)

In our cohort, blinking was elicited most frequently by stimulation of the uncus/amygdala, as well as the anterior fusiform and parahippocampal gyrus. The classical functional attribution to the fusiform gyrus is high level visual processing. In detail, previous electrical stimulation studies delineated circumscribed fusiform regions that are associated with face perception and perception of visual scenes (Parvizi et al. 2012; Mégevand et al. 2014). The parahippocampal gyrus, on the contrary, encodes for emotion processing, visuo-spatial navigation, and episodic memory (Kanwisher et al. 1997; Levy et al. 2001; Kirwan and Stark 2004; Smith et al. 2004; Mitterschiffthaler et al. 2007; Tendolkar et al. 2008). The parahippocampus seems to play an important role in encoding and retrieving contextual associations from a broad cerebral network (Bar et al. 2008; Aminoff et al. 2008, 2013; Peters et al. 2009; Norman-Haignere et al. 2012). This is substantiated by our tractography results, showing connections from parahippocampal regions to the limbic, frontal, pericentral, occipital, and cerebellar brain areas. Thus, one might speculate that the mesial temporal regions do not generate blinking, but modulate the frontal corticobulbar projections, while integrating emotional, visuospatial and sensorial input from the cortical and subcortical regions mentioned above (Fig. 3). In other words, also in humans the mesial temporal region probably adjusts the ipsilateral blinking based on its integrated contextual associations (Muakkassa and Strick 1979; Morecraft et al. 2004). Considering the isolated activation of a single muscle during ipsilateral blinking, an alternative conceivable hypothesis could be that the stimulated regions connect to a highly localized but elusive cortical area for ipsilateral blinking that connects with the ipsilateral primary motor cortex and facial nucleus via the ipsilateral cortico-spinal tract.

Ictal blinking has been described in epilepsy patients, most frequently with parieto-occipital and insulo-opercular seizure onsets and typically manifested bilaterally (Marchi et al. 2016; Lagarde et al. 2020; Peltola et al. 2020; Wang et al. 2020). Unilateral blinking only occurred in association with an activation of the mesial temporal lobe in a large-scaled SEEG study (Lagarde et al. 2020). Similarly, Benbadis et al. observed unilateral blinking during the first 37 s after the clinical onset of frontal or temporal lobe seizures (Benbadis et al. 1996), which most likely reflected the direct temporal activation or spread of the seizure activity towards the temporal lobe. Further, ictal blinking occurred only after a longer delay in occipital seizures compared to occipito-temporal seizures (Lagarde et al. 2020), potentially due to the longer propagation time towards the temporal lobe or precentral region. This is in line with our finding of an anterior temporal association of unilateral blinking as well as early stimulation experiments describing blinking following amygdaloid or mesial temporal stimulation (Lesser et al. 1985; Benbadis et al. 1996).

### Characteristics of Stimulated and Ictal Blinking

Electrical cortical stimulation induced a palpebral fluttering, which was tonic at higher stimulation amplitudes. Considering that the standard frequency of 50 Hz was used for electrical stimulation, the flutter/tonic eyelid contraction could be the clinical correlate of a ~ 50 Hz blinking, i.e. a high-frequency muscle contraction. This is in line with nerval stimulation studies showing a higher probability of a tonic motor response at higher stimulus frequencies (Begum et al. 2006; Raslan et al. 2017). Similarly, Maillard et al. reported different clinical motor responses in association with frequency changes of the EEG seizure pattern as well as during electrical intracranial stimulation with 1 Hz versus 50 Hz (Maillard et al. 2014). In two of our patients clonic unilateral blinking was observed after anterior temmporal stimulation close to the epileptogenic zone which triggered epileptic discharges. These epileptic discharges could also have elicited a clonic motor response; however, we also observed clonic blinking in three patients without after-discharges.

### Neuroanatomical Considerations

Most previous reports of blinking responses suggest an activation of cranial nerves as explanation for ictal or stimulated unilateral blinking. In case of stimulation with *subdural* electrodes, the facial motor response was thought to be caused by a direct activation of peripheral branches of the motor division of the facial nerve (Begum et al. 2006). Similarly, an ephaptic activation of dural branches of the trigeminal nerve by the fronto-temporal seizure activity was suggested (Sindou et al. 1994; Pestana and Gupta 2007; Jadhav et al. 2016). The so-called trigeminal-facial reflex, also known as blink reflex, might then lead to an ipsilateral face twitching (Livingston and Phillips 1957; Sindou et al. 1994; Gong et al. 2005b; Begum et al. 2006). However, in our patients, a direct activation of cranial nerves seems unlikely (1) because 3D reconstructions of the electrode positions showed clear parenchymal locations. (2) Further, altered sensation in the face would be expected in N. V activation, but was not observed in our patients. Similarly, Bartholow elicited unilateral blinking in one case by intracranial stimulation of the ipsilateral parieto-occipital region (Bartholow 1874), i.e. clearly outside of the primary motor region and distant from the meninges. In our patients, we thus suppose that blinking was facilitated via an activation of the supranuclear facial motor network rather than direct activation of cranial nerves, but this remains speculative. Future studies using low-frequency stimulation with direct measurement of the stimulus–response latency could provide further evidence but were not performed during the clinical evaluation.

Of note, the 16 patients with unilateral blinking encompassed significantly more female patients and more patients with temporal lobe epilepsy compared to the control group without blinking. The latter might be due to a selection bias, as more temporal SEEG electrodes were implanted and available for stimulation in patients with a supposed temporal epileptic zone. Nevertheless, it might also reflect known gender differences in emotion perception and processing including facial expression (Schulte-Rüther et al. 2008; Houstis and Kiliaridis 2009; Mcduff et al. 2017). Interestingly, previous functional MRI studies described an activation of a prefrontal-temporal-parietal network in the context of emotional perspective taking, with a stronger activation of the inferior frontal and superior temporal regions in women compared to men (Schulte-Rüther et al. 2008). To investigate this further, larger-scaled follow-up studies are needed.

## Limitations

Our study is limited by the fact that all our observations were made in patients with epilepsy whose brains might be functionally and structurally reorganized, especially in patients with extensive lesions and in case epileptic seizures or the underlying pathology started early in life. The mean age at disease onset was 18.50 ± 11.57 years of age in the cohort of 16 patients with unilateral blinking. However, in the 44 controls it was even slightly earlier (14.50 ± 7.97 years of age) and disease duration did not relevantly differ (13.06 ± 7.81 vs. 14.68 ± 12.69 in the control group). Thus, stimulation results obtained in epilepsy patients cannot be generalized without reservation. However, fundamental functions like blinking are typically not affect by this kind of reorganization. Like all evaluations with depth electrodes, our study’s findings are potentially confounded by propagation away from the site of electrical stimulation. This is, in particular, relevant in case of stimulation of electrode contacts within the cerebral white matter. The induced pathophysiological effects of the latter are not fully understood, but it is likely that the electrical current is conducted ortho- or retrogonally to remote (grey matter) areas which are causal to the observed clinical effects. In other words, the anatomical correlate of ipsilateral blinking in case of blinking induced by stimulation of cerebral white matter is not exactly at the electrode location but within the connectivity of the fibers. Our approach is further limited by the moderate spatial resolution. Driven by the clinical hypothesis and safety aspects, only as few as possible electrodes have been implanted, typically limited to one, i.e. the epileptic hemisphere. This entails that during ictal unilateral blinking the seizure pattern could have had already spread to the contralateral subcortical brain regions. In the simultaneous surface EEG-recording, though, no contralateral seizure pattern was recorded. Moreover, we could not evaluate the presumably important role of the occipital cortex, the basal ganglia and the reticular formation in unilateral blinking (Ebert et al. 1996; Smit 2009; Lagarde et al. 2020), as we did not perform sEEG recordings/stimulation of these regions. Bihemispheric sEEG evaluations of healthy human brains with standardized electrode locations would be of course scientifically desirable but are ethically not acceptable.

Although unlikely, a trigeminal input to the ipsilateral blinking has to be discussed. Neurographic measurements on the N. V or the N. VII were not performed. Thus, more patients have to be examined to consolidate our data and additional neurographic measurement and bilateral intracranial EEG-recording would be desirable.

## Conclusion

We hypothesize that unilateral blinking is associated with an ipsilateral anterior temporal activation. The identified regions, especially the parahippocampal and anterior fusiform gyrus, might have a modulating influence on ipsilateral blinking by integrating contextual information. This is the first study to present intracranial stimulation data on unilateral blinking combined with tractography results.

## Abbreviations

CT: Computed tomography
DTI: Diffusion-tensor-imaging
EEG: Electroencephalography
HARDI: High-angular resolution diffusion imaging
LPMCd: Dorsal lateral premotor cortex
LPMCv: Ventral lateral premotor cortex
MRI: Magnetic resonance imaging
sEEG: Stereo-EEG
TLE: Temporal lobe epilepsy

## Declarations

### Data Availability

The data that support the findings of this study are available from the corresponding author upon reasonable request.
